# Variant-beta luteinizing hormone is not associated with poor ovarian response to controlled ovarian hyperstimulation

**DOI:** 10.1186/1477-7827-12-20

**Published:** 2014-03-13

**Authors:** Hans I Hanevik, Hilde T Hilmarsen, Camilla F Skjelbred, Tom Tanbo, Jarl A Kahn

**Affiliations:** 1 Fertilitetsklinikken Sør, Telemark Hospital, Porsgrunn, Norway; 2 Department of Laboratory Medicine, Section of Medical Genetics, Telemark Hospital, Skien, Norway; 3 Department of Gynecology, Oslo University Hospital and University of Oslo, Oslo, Norway

**Keywords:** Luteinizing hormone, Genetic variant, Variant-beta LH, Ovarian response, IVF

## Abstract

**Background:**

The most common genetic variant of luteinizing hormone (LH), variant-betaLH, has a different bioactivity than the wildtype. Carrying the variant allele was associated with an increased consumption of exogenous gonadotropin to achieve optimal ovarian response for in vitro fertilization procedures (IVF). The aim of this study was to examine if variant-betaLH was also more common in patients with a poor ovarian response to exogenous gonadotropin which negatively influenced treatment outcome.

**Findings:**

36 patients with poor ovarian response to ovarian stimulation for IVF and 98 controls with a normal response were genotyped for variant-betaLH using DNA sequencing. The carrier frequency in the control group was 17%. No association was found between poor ovarian response and variant-betaLH.

**Conclusions:**

Testing patients for variant-betaLH prior to IVF is unlikely to predict poor ovarian response.

## Findings

### Background

The most common form of genetically determined variation in luteinizing hormone (LH) structure is variant-betaLH (v-betaLH) [1]. v-betaLH is caused by a doublet of single nucleotide polymorphisms (SNPs) in the *LHB* gene that induces a substitution of amino acids (LHB Trp8Arg and LHB Ile15Thr) in the beta subunit of LH [2]. The LHB 15Thr introduces an additional glycosylation site to v-betaLH, probably affecting the serum half-life of LH and thereby its bioactivity [3-5]. Various physiological and clinical implications of v-betaLH were proposed, including infertility and premature ovarian failure [6,7]. In the field of in vitro fertilization (IVF), patients who required an increased amount of recombinant follicular stimulating hormone (rFSH) to achieve an optimal ovarian response in controlled ovarian hyperstimulation (COH) were classified as hypo-responders [8]. Alviggi and co-workers reported that v-betaLH was more common in hypo-responders in a series of 60 Italian IVF patients [9]. This finding was recently confirmed in a larger series of Danish IVF patients [8], and is of interest when searching for genetic predictors of ovarian response to COH [10]. Poor ovarian response (POR) has similarities to hypo-response, but is more adverse for the patient’s treatment outcome [11]. Patients with POR have a markedly decreased chance of pregnancy from IVF treatment [12], whereas hypo-responders by definition achieve an optimal ovarian response [8]. The aim of the present study was to investigate if v-betaLH was more common in IVF patients with POR. If so, this would strengthen the case raised by Alviggi and co-workers for determining v-betaLH status in patients prior to COH [8].

## Methods

### Patients

Genomic DNA from 134 IVF patients was obtained from a preexisting biobank established to study genetic predictors of COH. Background data and data on other putative genetic predictors for ovarian response to COH in these patients were already published together with further details on patient recruitment and selection [13-18]. In brief all patients undergoing COH at Fertilitetsklinikken Sør from January 2003 to June 2009 were eligible for participation in the biobank. By feeding the criteria shown in Table 1 into the patient database, patients were retrospectively classified as having had a normal or poor ovarian response. The potential participants’ patient files were then checked manually for accuracy of information in the database, and a total of 338 patients were asked to contribute to the biobank by signing a consent form and delivering a blood sample. For all patients in the present study COH was by gonadotropic releasing hormone (GnRH) agonist mid luteal phase down regulation and rFSH (Puregon®, Merck, NJ, USA or Gonal-F®, Merck-Serono, Geneva, Switzerland). The standard starting dose of rFSH was 150 IU/day. Patients who were judged by clinicians to be at risk for POR had their starting dose adjusted without any specific standard of adjustment. During COH the ovarian response to COH was evaluated by ultrasound, and the dose of gonadotropin adjusted if necessary. Ovulation induction was by injection of urine-based or recombinant human chorionic gonadotropin (hCG) when at least one follicle had a mean diameter of 17 mm. Oocyte retrieval took place 34–36 hours after hCG injection. Available for the present study were 36 POR patients, and 98 controls with a normal ovarian response to COH.

**Table 1:** Inclusion- and exclusion criteria

	**Poor ovarian response**	**Controls**
**Inclusion-criteria:**		
*IU of rFSH per day*	150-200	150-200
*No. of oocytes obtained by oocyte retrieval*	≤ 3	5-13
**Exclusion-criteria:**		
*IU of rFSH per day*	< 150 or > 200	<150 or > 200
*Age*	> 40 years	> 40 years
*Other*	Polycystic ovary syndrome	As for poor ovarian response.
	Previous adnexal surgery	Present or previous signs of moderate or severe ovarian hyperstimulation syndrome
	Ovarian endometriosis or other ovarian tumour	
	Hypogonadism	
	Ovarian cysts > 3 cm	

### Genotyping

Genotyping was done by DNA sequencing. Genomic DNA was isolated from peripheral blood according to standard procedures using EZ1 BioRobot (QIAGEN) and stored at −20°C. Each PCR-reaction contained 50 ng of genomic DNA, 0.20 M of each primer, 1x AccuPrime PCR buffer II (Invitrogen), 0.60 U AccuPrime Taq DNA polymerase (Invitrogen) and milliQ H_2_O to a final volume of 12.5 l. Primer sequences used: LHB F 5′-GGG TGA AGC AGT GTC CTT GT-3′ and LHB R 5′-GAA GAG GAG GCC TGA GAG TT-3′ [2]. Cycling conditions were: 95C for 5 min, then 35 cycles of 95C for 30 s, 65C for 30 s and 72C for 30 s, then 72C for 10 min, then 4C ∞. The primers amplified most of exon 2 and 3 of *LHB* and gave a specific PCR-product excluding the homologous *CGB* genes (verified by gel electrophoresis and blast of sequence). The PCR-products were sequenced in both directions using BigDye Terminator kit v.3.1 (Applied Biosystems) and an ABI3130xl Genetic Analyzer (Applied Biosystems) according to the manufacturer’s procedures. The sequences were aligned to *LHB* RefSeq NG_011464.1 [19] using SeqScape v.2.6 software (Applied Biosystems). Samples were analysed in the order they arrived at the lab, often giving samples from both groups of patients in each run. Repeated sequencing of a random 16% subset yielded 100% identical sequences.

### Statistics

Sample size was limited to 36 cases and 98 controls by the study-design. Chi-square statistics were used for comparison of carrier and allele frequency of v-betaLH. Prior data indicated that the carrier frequency among controls was 0.11 [8]. If the true carrier frequency among cases was threefold, 0.33, then the null hypothesis that the carrier frequency for cases and controls were equal would be rejected with probability (power) 0.82 [20]. Other comparisons between groups were by student’s t-test or chi-square. For all comparisons differences between groups were considered statistically significant if reaching a p-value of <0.05.

## Results

Table 2 shows the clinical and genetic data for the two groups. The background data included in the table were already published [13-18]. LHB Trp8Arg and LHB Ile15Thr were in complete linkage disequilibrium and were denominated v-betaLH. There were no significant differences in carrier frequency or allelic frequency of v-betaLH between groups. The only homozygous carrier was in the control group.

**Table 2:** Clinical and genetic data

**Characteristic**	**Poor ovarian response (n = 36)**	**Controls (n = 98)**
*Age mean (95% CI)*	34.0^b^ (32.8-35.3)	32.4 (31.9-32.8)
*BMI (kg/m*^*2*^*) mean (95% CI)*	26.5^b^ (24.3-28.7)	23.8 (23.1-24.5)
*No. of patients of other ethnicities than Caucasian (%)*	3 (8.3)	4 (4.1)
*First time COH (%)*	32 (88.9)	83 (84.7)
*Early follicular phase s-FSH*^*a*^*mean (95% CI)*	6.0 (5.3-6.7)	5.8 (5.4-6.3)
*First dose of rFSH (IU) mean (95% CI)*	160 (152–168)	159 (155–162)
*Duration of rFSH medication (Days) mean (95% CI)*	10.8 (10.2-11.3)	10.9 (10.8-11.1)
*Total dose of rFSH (IU) mean (95% CI)*	1739 (1616–1863)	1738 (1692–1784)
*Oocytes retrieved mean (95% CI)*	1.5^b^ (1.1-2.0)	9.0 (8.5-9.5)
*No. of embryos transferred mean (95% CI)*	0.6^b^ (0.4-0.9)	1.6 (1.5-1.7)
*Live births (% of started COH)*	2^b^ (6)	45 (46)
*No. of patients with v-betaLH (%)*	4 (11)	17 (17)
*No. of alleles with v-betaLH (%)*	4 (6)	18 (9)

^a^Not registered for all patients. N registered for the respective groups: 32, 76.

^b^Statistically significant (p-value of <0.05) difference compared to controls by student’s t-test or chi square.

## Discussion

The present data are, to our knowledge, the first to estimate the prevalence of v-betaLH in a Norwegian population. A carrier frequency of 17% amongst normal responders to COH in an IVF population is comparable to 11% in a similar Danish IVF population [8]. In unselected populations a wide variation of v-betaLH carrier frequency was reported ranging from 42% in Finnish Lapps through 19% in Swedes to 7% in Hispanics in the USA [1]. No association was found between POR and v-betaLH in this study. This finding is in accordance with the only reported genome wide association study concerning ovarian response to COH [21]. Alviggi and co-workers [8] suggested that IVF patients with v-betaLH lack sufficient LH activity to adequately support FSH activity in multiple follicular development, leading to hypo-response to COH as defined above. The present results suggest that the decreased ovarian response in POR patients may require a different explanation, and point to the question of whether ovarian response to COH declines gradually from normal via hypo to poor, or if the three should be considered separate situations altogether [22]. The hypothesis proposed by Alviggi and co-workers [8], that carriers of v-betaLH could benefit from exogenous LH in their COH, seems unlikely to apply to POR patients from the present results; however, a different study design is required to test this properly.

Differences in numbers of oocytes retrieved, embryos transferred and live births were as expected from the inclusion- and exclusion criteria. There were no differences between groups regarding the FSH receptor SNPs reported to influence ovarian response to COH (data not shown). The control group was also not matched to the POR group, leading to differences in age and BMI between groups. Albeit small, these differences are a possible confounder in the study inasmuch as BMI was reported to influence ovarian response to COH [23] and the risk of POR increases with age [11]. The use of rFSH was similar between groups, reflecting that POR was unexpected by clinicians for the patients in the POR-group.

All COHs in this study, as in the studies by Alviggi and co-workers [8,9], were by GnRH agonist mid luteal phase down regulation. Whether v-betaLH is associated with hypo-response or POR in an antagonist protocol is unknown. However, the low endogenous level of LH in agonist cycles could arguably make v-betaLH more influential in agonist than antagonist cycles.

Genotype data from the same patients regarding other signaling systems of importance in ovarian physiology and their association with ovarian response to COH were presented earlier [13-18]. This raises the question of multiple testing in genetic association studies. However, as the present study showed negative results, concerns over false positive findings were unwarranted.

The present study had two main limitations; sample size and inclusion criteria. The above sample size calculations show that the study had adequate power to find a difference between groups if v-betaLH prevalence was three times higher in the POR group than in controls. A study with more patients could have detected smaller differences between groups. However, to avoid a high rate of false negatives when applying v-betaLH as a predictor of POR, a high prevalence of v-betaLH in the POR group is required.

The inclusion criteria for POR patients did not comply with ESHRE’s Bologna criteria [11]. The criteria for POR found in Table 1 were set to identify patients in which POR came unexpectedly, as it is in these patients that novel predictors of ovarian response could be most useful. Also the criteria in Table 1 assured that the patients included had few other known factors that could influence their ovarian response to COH apart from the putative genetic ones such as v-betaLH. The Bologna criteria on the other hand have ‘advanced maternal age’ and ‘previous POR’, as two of three criteria for POR, making them inadequate for identification of patients with an unexpected poor response, at least retrospectively as done in this study.

Other limitations in the present study were unavailability of s-LH measurements prior to and during COH, and no data on s-AMH or antral follicle count to predict ovarian response to COH.

## Conclusions

In conclusion this study found no association between v-betaLH and unexpected POR. This indicates that it is unlikely that testing IVF patients for v-betaLH prior to COH would contribute to predicting POR.

## Abbreviations

BMI: Body mass index; CI: Confidence interval; COH: Controlled ovarian hyperstimulation; ESHRE: European society for human reproduction and endocrinology; FSH: Follicular stimulating hormone; hCG: Human chorionic gonadotropin; IU: International units; IVF: In vitro fertilization; LH: Luteinizing hormone; LHB: Beta subunit of luteinizing hormone; LHB: Gene encoding beta subunit of luteinizing hormone; PCR: Polymerase chain reaction; POR: Poor ovarian response; rFSH: Recombinant follicular stimulating hormone; v-beta LH: Variant beta luteinizing hormone.

## Competing interests

The authors declare that they have no competing interests.

## Authors’ contribution

HIH participated in designing the study, was responsible for patient recruitment, was responsible for data analysis, drafted the manuscript. HTH participated in designing the study, assisted in patient recruitment, was responsible for collection and handling of blood samples, carried out the DNA sequencing, assisted in data analysis. CFS participated in designing the study. TT participated in designing the study. JK conceived of the study, was responsible for designing it, assisted in patient recruitment. All authors read and approved the final manuscript.
